# Does the Low-level occupational exposure to volatile organic compounds alter the seasonal variation of selected markers of oxidative stress? A case–control study in nail technicians

**DOI:** 10.1186/s12995-016-0125-6

**Published:** 2016-07-21

**Authors:** Peter Grešner, Radosław Świercz, Magdalena Beata Król, Ewa Twardowska, Jolanta Gromadzińska, Wojciech Wąsowicz

**Affiliations:** Department of Toxicology and Carcinogenesis, Nofer Institute of Occupational Medicine, 8, Sw. Teresy str., 91-348 Lodz, Poland

**Keywords:** Oxidative stress, Seasonal variation, Occupational exposure, Volatile organic compound, Nail technicians

## Abstract

**Background:**

In this study we tested whether the seasonal variations in levels of selected biomarkers of oxidative stress in female nail technicians occupationally exposed to low levels of volatile organic compounds (VOCs) differ significantly from those observed among healthy unexposed controls. Airborne levels of selected VOCs in nail salons were also analyzed and tested for associations with seasonal variations of the levels of biomarkers among nail technicians.

**Methods:**

The study enrolled 145 female nail technicians and 145 healthy unexposed female controls. The airborne VOCs and levels of biomarkers were assessed by GC-MS chromatography and absorption/fluorescence spectrophotometry, respectively.

**Results:**

Plasma levels of thiobarbituric acid reactive species, ceruloplasmin, the activity of glutathione peroxidase (GPx1) and the SOD1/GPx1 activity ratio presented significant differences between the so-called “hot” and “cold” seasons in the case of nail technicians as well as in unexposed controls (*p* < <0.0001 for all four biomarkers). The pattern of these variations among nail technicians was found to be significantly different compared to that of the control subjects (*p* < <0.0001). Although such differences might intuitively be attributed to occupational exposure of nail technicians to VOCs, which was found to be higher during the “cold” season compared to the “hot” one, our study provided only limited evidence in favor of the hypothesis, that the different pattern of seasonal variations of biomarkers among nail technicians might have resulted from seasonal fluctuations in their occupational exposure to VOCs.

**Conclusion:**

Further investigation is thus needed in order to elucidate the effect of low-level occupational exposure to VOCs on seasonal variations of biomarkers of oxidative stress.

**Electronic supplementary material:**

The online version of this article (doi:10.1186/s12995-016-0125-6) contains supplementary material, which is available to authorized users.

## Background

An increasing amount of evidence is available suggesting that prolonged occupational exposure to volatile organic compounds (VOCs; including ethanol, acetone, toluene or xylene) may result in increased levels of biomarkers of oxidative stress, DNA damage and dysregulation of cellular antioxidant defense and trigger processes leading possibly to carcinogenesis [1–8]. It is, however, a bit astounding, that some studies report these adverse outcomes to be observable also if no biological and occupational exposure limits are being exceeded [9, 10].

Licensed nail technicians present an occupational group which has raised in number recently due to increased popularity of nail salon services throughout the western countries. Illustratively, the number of nail technicians registered in USA has increased by 345 % since 1991 reaching over 393.000 in 2005 [11]. Services provided by these technicians involve a number of chemical products comprising toxic substances including VOCs. The mixture of VOCs most frequently found in nail salons consist predominantly of ethanol, acetone, toluene, xylene and several acetates. The International Agency for Research on Cancer (IARC) has classified ethanol as a group I carcinogen and exposure to this compound has been recognized as a strong risk factor for several forms of cancer. The ethanol-induced carcinogenesis involves induction of oxidative stress, as one of possible mechanisms [12]. On the other hand, acetone, toluene and xylene have not been classified by IARC as neither carcinogenic nor genotoxic to humans, but evidence on increased levels of markers of oxidative stress, chromosomal aberrations or cancer incidence among workers occupationally exposed to these substances, even at exposures far below occupational limits, has been growing recently. Although precise mechanisms underlying these effects are currently unknown, the role of oxidative stress is anticipated, as well (see [13]).

Recent studies report various adverse health-related effects of occupational exposure to chemical products used in nail industry, including skin, eyes and upper airways mucosa irritation [11, 14–16], headaches [16, 17], musculoskeletal and reproductive system disorders [11, 15–19] and asthma [11, 14]. Nevertheless, despite the undoubted occurrence of VOCs in such occupational exposure, based on available studies it rather cannot be assumed that nail technicians are being overexposed to VOCs [20]. On the other hand, the use of chemical products in close vicinity to technician’s breathing zone and eyes during working procedures may constitute a source of considerable health risks. Occupational exposure limits (OELs) for chemical substances in Poland are set by Central Institute for Labor Protection – National Research Institute in Warsaw. Values of the most frequently used forms of OEL, the time-weighted average (TWA)-OEL, defining an average exposure to a substance during a 8-h workday, can be retrieved from the Institute’s online archive [21]. However, in our recent study we have shown that despite the low level of occupational exposure to VOCs, far below the respective TWA-OELs, nail technicians may be subject to dysregulation of the levels of selected biomarkers of oxidative stress and DNA damage (increased activities of glutathione peroxidase 1 and ceruloplasmin, increased ratio of glutathione peroxidase 1 and superoxide dismutase 1, decreased level of oxidatively-generated DNA damage) [13]. Intriguingly, studies on human subjects, as well as those performed upon animal models, seem to suggest, that the levels of biomarkers of oxidative stress present a distinct seasonal patterns of variations, with the summer season being associated with significantly higher levels compared to the winter one [22–30].

In this study we aimed to investigate whether the levels of selected markers of oxidative stress in nail technicians occupationally exposed to VOCs present any kind of seasonal variation and if so, whether these changes differ significantly from those found in general control population. To this end, we evaluated a panel of biomarkers of oxidative stress (the plasma levels of thiobarbituric acid-reactive species (TBARS) and ceruloplasmin (Cp), the blood plasma activity of glutathione peroxidase (GPx3), the red blood cells’ activities of GPx1, zinc-copper superoxide dismutase (SOD1) and the SOD1/GPx1 ratio) in female nail technicians working in nail salons during the late spring/summer (the so-called “hot”) and late autumn/winter (the so-called “cold”) season and test their differences against healthy unexposed controls. Moreover, airborne concentrations of selected organic solvents in workrooms of respective nail salons were measured and analyzed in order to find out whether these concentrations change throughout the year and whether they might be associated with possible seasonal variations of the levels of biomarkers in nail technicians.

## Methods

### Recruitment of study and control subjects

The study group consisted of nail technicians occupationally exposed to low levels of VOCs, recruited from nail and/or beauty salons providing manicure and/or artificial nail sculpturing services selected randomly within downtown as well as suburb areas of the city of Lodz in central Poland. In total, three hundred and eighteen nail/beauty salons were contacted during the recruitment. Ultimately, the study group consisted of 145 female nail technicians of white descent aged 21 to 64 years (median age at the time of enrollment 34 years; interquartile range (IQR): 27–42 years.) from 109 different nail and/or beauty salons. All enrolled nail technicians were scheduled for blood withdrawal and workroom air sampling. If any health-related problems happened to occur to nail technicians on scheduled days, other days were scheduled to ensure all enrolled nail technicians were healthy at the time of measurements.

The control group consisted of 145 healthy female volunteers, aged 19 to 59 years (median age at the time of enrolment 33 years; IQR: 27–42 years), who agreed to undergo examinations. All control subjects were also of white descent, citizens of the Lodz district, without any history of occupational exposure to organic solvents or airborne acrylates, not involved in nail industry.

Both the nail technicians and controls were enrolled in two waves: the first one was between May and July (i.e., the late spring/summer period, hereinafter referred to as the so-called “hot” season”) involving 66 nail technicians from 45 different nail salons (median age: 33 years; IQR: 28–41 years) and 101 control subjects (median age: 32 years; IQR: 26–42 years), and the other one between November and February (i.e., the late autumn/winter period, hereinafter referred to as the so-called “cold” season”) involving 79 nail technicians from 64 different nail salons (median age: 34 years; IQR: 27–42) and 44 controls (median age: 34 years; IQR: 27–42 years). Supplementary information on lifetime tobacco-smoking history was collected from each individual enrolled in the study. All individuals were classified as never- or ever-smokers based on the criterion suggested by Pomerleau et al. [31], according to which the ever-smokers class consisted of current or former smokers with the lifetime history of smoking higher than 19 cigarettes. Additionally, precise lifetime tobacco consumption was expressed by means of the pack-years (i.e., the number of cigarette packs smoked per day times the number of years as a smoker; PY). The summary characteristics of both groups involved in the study is provided in Table 1.

**Table 1:** Characteristics of the groups of subjects involved in the study

	“Hot” season (May-July)		“Cold” season (November-February)	
Feature	Nail Technicians	Controls	Nail Technicians	Controls
Total number	66	101	79	44
Age [years]	33 [28–41]	32 [26–42]	34 [27–42]	34 [27–42]
Smoking status [never/ever]	26/40 [0.39/0.61]^*^	69/32 [0.68/0/32]	42/37 [0.53/0.47]	29/15 [0.66/0.34]
Pack-years	0.15 [0.0–22.5]^**^	0.0 [0.0–25.0]	0.0 [0.0–13.5]	0.0 [0.0–3.8]

Numerical data for age and pack-years presented as median [range]. Smoking status [never/ever-smokers] expressed as absolute [relative] counts. Age and pack-years were tested for significant differences among the groups using the Kruskal-Wallis *H* test with subsequent post-hoc comparisons performed using the *z* test. Smoking status was tested for significant differences among the groups using the log-linear analysis of contingency tables

^*^ *p* < 0.0005 (nail technicians vs. control subjects during the “hot” season)

^**^ *p* < 0.01 (nail technicians vs. control subjects during the “hot” season)

### Ethical issues

Prior to any further measurements, written and informed consent for participation in this study was obtained from each participant. All procedures performed in this study were in accordance with the ethical standards of the institutional and/or national research committee and with the 1964 Helsinki Declaration and its later amendments or comparable ethical standards. The study was approved by the Bioethics Committee of the Nofer Institute of Occupational Medicine (resolution no. 5/2011).

### Blood withdrawal

To avoid the effect of daytime itself on the oxidative stress status, blood withdrawal was performed always in the morning before work, at around 8:00 – 9:00 am. A sample of peripheral blood was collected from each participant involved in the study by venipuncture into tubes containing either EDTA or heparin as anticoagulants. Heparinized blood was centrifuged (10 min, 1500 × g, 4 °C) in order to isolate blood plasma and red blood cells (RBCs). Further on, blood plasma was frozen at −80 °C and subsequently used in order to determine the level of TBARS, the activity of GPx3 and plasma level of Cp. In lysed RBCs the GPx1 and SOD1 activity assays were performed.

### Assays of biomarkers of oxidative stress

The whole panel of biomarkers of oxidative stress was assayed in each subject enrolled in the study.

The blood plasma level of TBARS was determined using the method previously described by Wasowicz et al., based on the reaction of 2-thiobarbituric acid (TBA) with malondialdehyde, which is a naturally occurring side-product of lipid peroxidation [32]. The assay was conducted on a PerkinElmer LS50-B fluorescence spectrometer (PerkinElmer, Shelton, CT, USA) and the concentration of TBARS in blood plasma was expressed in μM.

The activity of RBCs’ SOD1 was determined spectrophotometrically according to method described earlier by Beauchamp and Fridovich. The method consists in SOD1-mediated inhibition of the reduction of nitro blue tetrazolium (NBT) and the enzymatic activity of SOD1 was expressed in units per mg haemoglobin (U/mg Hb) with 1 unit corresponding to amount of enzyme that reduces the rate of NBT reduction to 50 % per 1 min [33].

The GPx activities in peripheral blood plasma (GPx3) and RBC (GPx1) were determined according to method described by Paglia and Valentine based on spectrophotometric assessment of the oxidation of NADPH in the presence of exogenous glutathione reductase, NADPH, utilizing *tert*-butyl hydroperoxide as a substrate [34]. The enzymatic activity was expressed in units per milliliter (U/mL) and unit per gram of haemoglobin (U/g Hb) for plasma and RBC, respectively, with 1 unit equal to 1 μmol NADPH oxidized per 1 min.

Oxidase activity of ceruloplasmin in blood plasma was determined according to method described earlier by Sunderman and Nomoto [35]. The method consists in Cp-mediated oxidation of *p-*phenylenediamine hydrochloride yielding a color product measured spectrophotometrically at 535 nm. The rate of formation of the color product, corrected for nonenzymatic oxidation of *p-*phenylenediamine hydrochloride, is proportional to the blood plasma concentration of Cp, expressed in g/l.

All assays were optimized and performed using a Thermo Scientific Evolution 3000 UV/VIS spectrophotometer (Thermo Scientific, Wlatham, MA, USA).

### Air sampling and chromatographic determination of airborne solvents

Air samples from nail salon workrooms were collected using the Sensidyne GilAir-3SC EX Atex air sampling pumps (Sensidyne, Clearwater, FL, USA) equipped with Anasorb 747 sorbent tubes (SKC, Dorset, UK). All measurements were performed as stationary but in the case of each nail technician the sampling pump was placed on a working table directly within the nail technician’s working field, close to her breathing zone, in order to assure that the collected air sample mimicked the air the nail technician was breathing during the treatment. Each air sampling was performed on a weekday in busy hours during a period in which nail treatments were scheduled and lasted from 41 to 234 min (median 116 min; IQR 89–142 min). A single workroom air sample was collected per salon. If there were more nail technicians in one salon enrolled in the study, air sampling was scheduled on a day when all of them were at work and performing nail treatments. Prior to each sampling, sampling pumps were calibrated using a soap bubble flow meter and were operated during sampling at flow rate of 100 mL/min. After sampling, the tubes were stored at −20 °C prior to desorption and analysis which were performed according to method described previously by Gjolstad et al. [36] with minor modifications. Briefly, solvent tubes were desorbed in 1.5 mL of carbon disulfide and N,N-dimethylformamide (98:2, v/v) and sonicated for 30 min. Airborne solvents were quantified by means of external standard calibration with volumetrically prepared solution in carbon disulfide and dimethylformamide. Sorbent from an unexposed tube was added in order to correct for desorption efficiency. Chromatographic determination of solvents in workroom air samples was performed using Agilent’s 6890 N gas chromatograph with 5973 Network mass selective detector and HP-5MS capillary column (Model 19091S-433; 30 m × 0.25 mm × 0.25 μm was used (Agilent Technologies, Palo Alto, CA, USA). In all analytical measurements, helium was used as carrier gas. The column temperature was initially held at 35 °C for 3 min, raised at 20 °C/min to 80 °C and then held at 80 °C for 7 min and finally raised at 5 °C/min up to 150 °C. The total runtime was 26.25 min. Mass spectrometer operated in SCAN mode with an associated m/z range set from 40 to 250. Peak integration was based on extracted ion and retention times for standard. All chemicals used in chromatographic assays except for ethanol (Witko, Poland), acetone, N,N-dimethylformamide (both from Polish Chemicals, Poland) and carbon disulfide (Analityk, Poland) were from Sigma-Aldrich (USA).

### Statistical analysis

Normal distribution of numerical data was tested by means of the Shapiro-Wilk *W* test. Since the data on levels of biomarkers of oxidative stress met all relevant criteria for normal distribution but were found to be significantly right-skewed, they were logarithmically transformed prior to further analysis. Transformed data fulfilling all the criteria for normal distribution are presented as mean ± standard deviation (SD) and expressed in ln-units. Analysis of outliers was performed on the basis of estimated Cook’s distances. Inter-seasonal and between-group differences in biomarkers of oxidative stress were tested for significance employing the multivariate analysis of covariance (MANCOVA) with the Wilk’s Lambda statistics. Univariate ANCOVA was also performed for each individual biomarker of oxidative stress. All analyses of covariance were adjusted to age and smoking-habits as confounders, but employing two different approaches: the analyses either involved (1) each subject’s age and lifetime pack-years as confounders in order to control for these additional two factors likely affecting the resultant oxidative stress, or (2) were performed following the stratification of all enrolled subjects according to their smoking status (i.e., never- or ever-smokers) involving only subject’s age as confounder in each stratum analyzed separately. Post-hoc univariate comparisons were performed using the Scheffé test.

Measured airborne concentrations of organic solvents departed from normal distribution and are thus provided as medians [range]. Between-season differences in airborne levels of organic solvents were tested for significance using the Mann–Whitney *U* test. Assessment of the combined exposure to the mixture of organic solvents with similar toxicological effects was performed using the American Conference of Governmental Industrial Hygienists (ACGIH) formula for evaluating the additive effect by means of the formula *Σ*(*C*_*i*_/*N*_*i*_), where *C*_*i*_ and *N*_*i*_ are the measured airborne concentration and time-weighted average occupational exposure limit (TWA-OEL) for *i*^*th*^ component. Polish TWA-OELs were used for the purposes of this study.

Simple inter-group comparisons of non-paired data were performed using the Mann–Whitney *U*, Student’s *t*, or two-sided exact mid-P test, depending on type and distribution of data. Comparison of more than two groups were performed using log-linear analysis of contingency tables (discrete data) or the Kruskal-Wallis *H*-test (continuous data). The associations between airborne levels of organic solvents and biomarkers of oxidative stress were assessed by partial correlations analysis (forward stepwise regression) and expressed by means of semi-partial correlation coefficients *r*_*sp*_.

All statistical calculations were performed using the Statistica 10 software package (StatSoft, Tulsa, USA).

## Results

### Seasonal variation in the levels of biomarkers of oxidative stress in nail technicians and controls

**Fig. 1:**
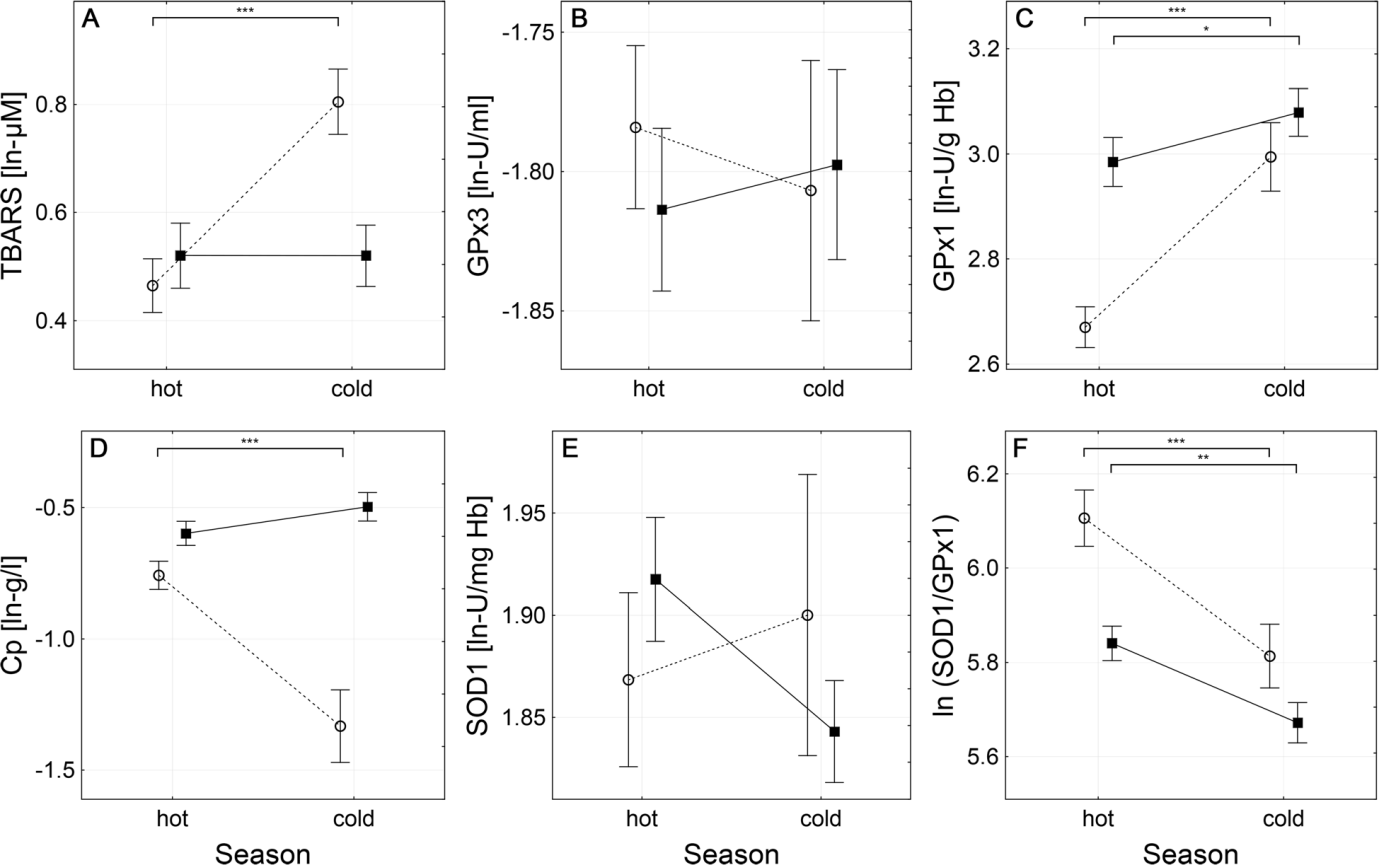
Levels of biomarkers of oxidative stress in nail technicians (solid line, ■) and control subjects (dashed line, ○) during the “hot” and “cold” season. Individual panels show data for the plasma levels of TBARS (**a**), the activity of GPx3 (**b**), the activity of GPx1 (**c**), the plasma level of Cp (**d**), the activity of SOD1 (**e**) and the SOD1/GPx1 activity ratio (**f**). All data are presented as *ln*-transformed means (central points) ± SD (error bars). Natural-base logarithm transformation was employed in order to normalize the distribution of raw data for the purpose of MANCOVA analysis. Asterisks indicate the respective levels of significance of inter-season differences observed among nail technicians and/or control subjects. The activity of SOD1 (panel **e**) was found to be significantly affected by the interaction between the season and the group of given individual (*p* < 0.05) although no significant inter-seasonal differences were found * *p* < 0.05; ** *p* < 0.0005; *** *p* < < 0.0001

Seasonal variations in age-and-PY-adjusted levels of biomarkers of oxidative stress in nail technicians and control subjects are presented in Fig. 1. The multivariate analysis revealed the season, the group and their mutual interaction as factors significantly affecting the levels of investigated biomarkers of oxidative stress (*p* < <0.0001 for all three factors). Further univariate analyses revealed significant seasonal variations in four out of six investigated biomarkers of oxidative stress: blood plasma levels of TBARS and Cp, RBC’s activity of GPx1, and the ln-ratio of SOD1/GPx1 (*p* < <0.0001 (the main effects) for all biomarkers). Furthermore, the season and the group were found to interact significantly with each other in the case of all these biomarkers (the levels of significance for the group/season interaction: *p* < <0.0001 in the case of TBARS, GPx1 and Cp; *p* < 0.05 in the case of the ln-ratio of SOD1/GPx1) indicating, that the seasonal variations in investigated biomarkers of oxidative stress differ significantly between the two groups involved.

In fact, the blood plasma levels of TBARS (Fig. 1a) were found to be significantly increased during the “cold” season compared to the “hot” one in controls (“hot” vs. “cold” season: 0.46 ± 0.25 vs. 0.80 ± 0.20 ln-μM, *p* < <0.0001) but not in nail technicians, in the case of which no significant differences between the two seasons were observed (“hot” vs. “cold” season: 0.52 ± 0.24 vs. 0.52 ± 0.25 ln-μM, *NS*). In contrast to the “hot” season, during the “cold” one, the levels of TBARS in controls differed significantly from that of nail technicians (*p* < <0.0001).

In the case of the RBC’s GPx1 (Fig. 1c), the “cold” season was associated with significantly increased activities of this enzyme in both the controls (“hot” vs. “cold” season: 2.67 ± 0.20 vs. 2.99 ± 0.21 ln-U/g Hb, *p* < <0.0001) and nail technicians (“hot” vs. “cold” season: 2.98 ± 0.19 vs. 3.08 ± 0.20 ln-U/g Hb, *p* < 0.05). Although the levels of GPx1 differed significantly between controls and nail technicians during the “hot” season (*p* < <0.0001), the much more pronounced increase observed in controls resulted in the level of GPx1 activity reaching almost the one found in nail technicians, without any significant differences between these two groups during the “cold” season.

Concerning the blood plasma levels of ceruloplasmin (Fig. 1d), the seasonal changes between “hot” and “cold” season were observed only among controls with the “cold” season being associated with significantly lower values compared to the “hot” one (“hot” vs. “cold”: −0.76 ± 0.27 vs. -1.33 ± 0.46 ln-g/L, *p* < <0.0001), but not among nail technicians in the case of which the ceruloplasmin levels were slightly but insignificantly increased during the “cold” season (“hot” vs. “cold”: −0.60 ± 0.19 vs. -0.50 ± 0.24 ln-g/L, *NS*). Nail technicians presented increased blood plasma levels of ceruloplasmin compared to control subjects, irrespective of the season (“hot”: *p* < 0.01; “cold”: *p* < <0.0001).

In the case of the ln-ratio of SOD1/GPx1 (Fig. 1f), the seasonal variability was observed in both the controls (“hot” vs. “cold”: 6.11 ± 0.30 vs. 5.81 ± 0.22, *p* < <0.0001) as well as nail technicians (“hot” vs. “cold”: 5.84 ± 0.15 vs. 5.67 ± 0.19, *p* < 0.0005) with the “cold” season being associated with significantly lower values. Nevertheless, the difference was much more pronounced among controls. The value of the SOD1/GPx1 ln-ratio was significantly lower compared to control subjects, irrespectively of the season (“hot”: *p* < <0.0001; “cold”: *p* < <0.05).

In the case of the RBC’s activity of SOD1, no significant main effect of either the season or the group to which the given subject belonged was observed, although the resultant level of this biomarker was found to be affected by significant interaction between these two main factors (*p* < 0.05). Based on data presented in Fig. 1e it is obvious that inter-seasonal changes in SOD1 activity, although insignificant, differ in their direction, with the “cold” season resulting in slight reduction of SOD1 activity among nail technicians (“hot” vs. “cold”: 1.92 ± 0.12 vs. 1.84 ± 0.11 ln-U/mg Hb, *NS*), but slight increase among controls (“hot” vs. “cold”: 1.87 ± 0.22 vs. 1.90 ± 0.23 ln-U/mg Hb, *NS*), instead. Nevertheless, none of these inter-seasonal, as well as none of the between-group changes, reached the level of statistical significance.

The blood plasma activity of GPx3 (Fig. 1b) was found to be independent of the season in both the controls and the nail technicians, with no significant differences between these two groups being observable, either.

Individual’s smoking status was not found to interact significantly with the season and further modulate the above described seasonal changes in TBARS, GPx1, Cp, SOD1 and SOD1/GPx1, or the lack of changes in the case of GPx3: the pattern of seasonal changes observed among controls and nail technicians enrolled in this study was found to be the same in both the never-smokers and ever-smokers stratum, irrespective of whether univariate or multivariate analysis of covariance was employed (*data not shown*).

### Airborne levels of organic solvents in nail salon workrooms during the “hot” and “cold” seasons

The measured airborne concentrations of organic solvents in air samples from nail salon workrooms are summarized in Table 2. During the “cold” season, total pool of organic solvents in nail salon workrooms as well as the combined exposure of nail technicians to mixture of organic solvents were significantly higher compared to the “hot” one (24.22 mg/m^3^ vs. 7.60 mg/m^3^, *p* < <0.0001 for total measured pool; 0.057 vs. 0.019, *p* < 0.0005 for combined exposure). During both seasons, ethanol, 2-propanol and ethyl acetate were the three most abundant organic solvents found in air samples from the nail salon workrooms and, moreover, were found to be also significantly increased during the “cold” season compared to the “hot” one (*p* < <0.0001, *p* < 0.01 and *p* < 0.05, respectively). Intriguingly, toluene and isopropyl acetate were found to be significantly lower during the “cold” season (*p* < 0.05 and *p* < <0.0001, respectively), yet these organic solvents were found to be two orders of magnitude less abundant in workroom air samples, and thus their inter-seasonal differences were far less pronounced. The inter-seasonal differences in airborne levels of acetone, 2-butanone and n-butyl acetate were insignificant. Nevertheless, median airborne levels of all organic solvents analyzed in this study were found to be far below the respective Polish TWA-OELs.

**Table 2:** Airborne concentrations of organic solvents in nail salons, combined occupational exposure of nail technicians during the “hot” and “cold season

	TWA-OEL	“Hot” season	“Cold” season
Ethanol [mg/m^3^]	1900	1.57 [0.06–16.12]	6.96 [0.35–54.77]^****^
Acetone [mg/m^3^]	600	0.81 [0.03–43.23]	0.57 [0.05–61.69]
Toluene [mg/m^3^]	100	0.02 [0.004–0.30]	0.01 [0.000–0.62]^*^
2-propanol (isopropyl alcohol) [mg/m^3^]	900	1.31 [0.11–21.30]	3.44 [0.12–37.70]^**^
2-butanone (methyl ethyl ketone) [mg/m^3^]	200	0.04 [0.007–0.92]	0.05 [0.002–1.63]
Ethyl acetate [mg/m^3^]	200	1.36 [0.07–27.28]	3.96 [0.03–44.54]^*^
Isopropyl acetate [mg/m^3^]	600	0.02 [0.010–0.10]	0.01 [0.000–0.22]^****^
n-butyl acetate [mg/m^3^]	200	0.23 [0.021–4.87]	0.17 [0.00–18.53]
Total [mg/m^3^]	-	7.60 [0.57–70.62]	24.22 [1.55–132.28]^****^
Σ(C_i_/N_i_)	1.0	0.019 [0.002–0.170]	0.057 [0.002–0.254]^***^

Airborne levels of organic solvents measured in air samples collected from nail salon workrooms during the “hot” (*N* = 45) and the “cold” (*N* = 64) season. Data presented as median [range] airborne concentrations were tested for significant inter-seasonal differences using the Mann–Whitney *U* test. TWA-OEL stands for the Polish time-weighted average occupational exposure limit of the respective organic solvent. Σ(C_i_/N_i_) stands for the ACGIH measure of the additive effect of combined exposure of the mixture of organic solvents, with respective threshold value equal 1

^*^ *p* < 0.05; ^**^ *p* < 0.01; ^***^ *p* < 0.0005; ^****^ *p* < 0.0001

### Correlation analysis

Results of stepwise partial correlation analysis, in which associations between airborne levels of organic solvents and biomarkers of oxidative stress (adjusted for age distribution) were analyzed, are presented in Table 3. The activity of cytosolic GPx1 was positively correlated with airborne ethanol (r_SP_ = 0.26, *p* < 0.05), but negatively with airborne toluene (r_SP_ = −0.25, *p* < 0.05) levels. Moreover, the blood plasma concentration of Cp correlated positively with airborne ethyl acetate (r_SP_ = 0.22, *p* < 0.05), but negatively with isopropyl acetate (r_SP_ = −0.25, *p* < 0.05). The activity of RBC’s SOD1 was negatively correlated with airborne acetone (r_SP_ = −0.25, *p* < 0.05).

**Table 3:** Results of partial correlation analysis between airborne levels of organic solvents and biomarkers of oxidative stress in nail technicians

	TBARS [ln-μM]	GPx3 [ln-U/mL]	GPx1 [ln-U/g Hb]	Cp [ln-g/L]	SOD1 [ln-U/mg Hb]
Ethanol [ln-mg/m^3^]	0.1813	0.0677	**0.2628** ^*^	−0.1064	0.1004
Acetone [ln-mg/m^3^]	−0.0661	0.0915	−0.0567	0.2263	−**0.2537** ^*^
Toluene [ln-mg/m^3^]	−0.0147	−0.1793	−**0.2519** ^*^	−0.1047	−0.0036
2-propanol [ln-mg/m^3^]	−0.0583	−0.0456	0.0085	0.0543	0.1263
2-butanone [ln-mg/m^3^]	0.0589	0.0219	−0.0473	0.0380	0.1546
Ethyl acetate [ln-mg/m^3^]	−0.1057	0.1658	−0.0081	**0.2208** ^*^	0.0110
Isopropyl acetate [ln-mg/m^3^]	0.0080	0.0431	−0.0513	−**0.2522** ^*^	0.2146
n-butyl acetate [ln-mg/m^3^]	0.1906	0.0407	−0.1070	−0.0057	−0.1063

Presented are the semi-partial correlation coefficients (r_sp_) for each given pair of airborne level of organic solvent and level of analyzed biomarker. Forward stepwise regression procedure upon *ln*-transformed data was employed. Significant associations are indicated in bold with respective level of significance indicated by the superscript. The portion of variability explained by the association: ethanol vs. GPx1: 6.9 %; acetone vs. SOD1: 6.4 %; toluene vs. GPx1: 6.3 %; ethyl acetate vs. Cp: 4.9 %; isopropyl acetate vs. Cp: 6.4 %

^*^ *p* < 0.05

## Discussion

In this study, we investigated the seasonal variations in selected biomarkers of oxidative stress (the plasma levels of TBARS and Cp, activities of GPx3, GPx1 and SOD1, and the SOD1/GPx1 ratio) in the group of nail technicians occupationally exposed to VOCs and unexposed control subjects. Significant differences between the so-called “hot” and “cold” seasons were observed in the case of plasma levels of TBARS and Cp, the activity of GPx1 and the SOD1/GPx1 ratio, suggesting that these biomarkers of oxidative stress may be subject to seasonal variations. More importantly, statistical analysis revealed a significant interaction between the effect of the season and the occupational exposure, as we found the pattern of these variations among nail technicians to be different compared to that of the control subjects. In the “cold” season, the plasma levels of TBARS were found to increase significantly compared to the “hot” season, but only among controls with no changes being observable among nail technicians. In both investigated groups, the “cold” season was associated with increased activity of GPx1 compared to the “hot” one, nevertheless, the difference was more pronounced among controls. In the case of Cp, its plasma level in the “cold” season was found to drop significantly among controls, but was found to be significantly increased among nail technicians with the amplitude of inter-seasonal change being several times less the one observed among controls. Finally, the SOD1/GPx1 ratio was found to drop significantly in the “cold” season in both examined groups when compared to the “hot” one, nevertheless, the difference was more pronounced among controls. The activity of SOD1 seems to present opposite directions of changes due to seasonal variations in the two groups, although no significant changes for this biomarker were found.

In the literature, a couple of studies involving human subjects [22, 23, 25, 37] as well as animal models [22, 26–30] investigating the seasonal variability of the levels of biomarkers of oxidative stress can be found, suggesting that the summer (i.e., the “hot”) season is likely associated with increased levels of biomarkers of oxidative stress (including the plasma level of TBARS, the activities of antioxidant enzymes, the level of DNA damage) compared to the winter (i.e., the “cold”) one. Although the precise mechanism of such seasonal variability remains poorly understood, authors suggest that the balance may be shifted towards increased oxidative stress during the summer (i.e., the “hot”) season due to an increased sunlight radiation and/or increased ambient temperature and humidity during this season [23, 28, 37]. Endothelial activation [22], altered melatonin levels [25], intensified temperature-dependent lipid peroxidation due to altered iron content in certain proteins [38], altered levels of glucocorticoids and altered ADP sensitivity and oxidative capacity of mitochondria associated with circannual changes in metabolism [30] are all among proposed molecular mechanisms leading plausibly to such shift towards increased oxidative stress. In the present study, we did not observe such consistently increased levels of biomarkers of oxidative stress during the “hot” season among the control subjects. During the “hot” season, increased activity levels were found only for plasma ceruloplasmin and plasma GPx3, with the latter one showing only slight and insignificant change compared to the “cold” season. On the other hand, plasma levels of TBARS and the activity of GPx1 were significantly decreased during the “hot” season. It is somehow puzzling why the seasonal variation in biomarkers of oxidative stress observed in our study presented a pattern which seems to be incompatible with the patterns of variation reported previously by the above cited studies. Thorough survey of the currently available literature, however, revealed, that in available studies there is also a certain degree of inconsistency, as reports on significantly lower levels of activities of antioxidant enzymes or lack of any changes in plasma TBARS during the summer season compared to the winter one can be found [22, 28, 29]. For example, the changes observed in the group of control subjects involved in studies by Rossner et al. [39, 40] also seems not to be in line with the hypothesis that the summer season is associated with increased levels of the biomarkers: comparing the levels of biomarkers of oxidative stress and oxidatively generated damage to lipids and DNA measured during the three consecutive seasons (winter, summer, winter), among control subjects, a trend of gradual increase of the values was observed, instead of the summer-season values being significantly higher compared to the values from previous and subsequent winter seasons. It might thus implicate that some other factors, not considered in the hereby cited studies, are involved in the modulation of the resultant levels of biomarkers. As suggested by Moller et al., such plausible factors may include average daily influx of sunlight during a certain period (around 50 days) prior to sample withdrawal, with the highest importance being attributed to the average sunlight influx during last 3–6 days prior to the measurement [23]. No such parameter was controlled or monitored in any of the above mentioned studies, neither was it in the hereby presented study, what may, at least to some degree, explain the observed discrepancies between the studies. Moreover, it has to be kept in mind, that TBARS, for example, is not a malondialdehyde-specific biomarker and that this assay may in principle detect any thiobarbituric acid-reactive compound, not necessarily derived from lipid peroxidation. Nevertheless, this aspect is surely worth thorough examination.

More importantly, examining the outcomes obtained in this study, it seems plausible that the seasonal variations in biomarkers of oxidative stress among nail technicians occupationally exposed to VOCs present markedly different patterns when compared to those observed among control subjects. At first glance, it seems apparent, that amplitude of inter-seasonal changes of the plasma levels of TBARS, the activity of GPx1, the plasma levels of Cp and the SOD1/GPx1 ratio in nail technicians observed in our study was attenuated to some degree, reaching only 0.1 %, 29.1 %, 17.7 % and 57.7 % of the respective amplitudes observed among unexposed controls. Moreover, the direction of inter-seasonal changes of plasma levels of Cp and the activity of SOD1 among nail technicians seems to be reversed compared to the one observed among controls. It seems reasonably to state, that the pattern of inter-seasonal changes of these biomarkers observed in nail technicians is not only significantly different compared to the one observed among control subjects enrolled in this study, but it seems to be different also compared to the pattern of seasonal variations reported in the previously published studies cited above.

At this point, one would probably consider as what might be the plausible causes of such different patterns of seasonal variability of investigated biomarkers among nail technicians when compared to unexposed controls. Undoubtedly, occupational exposure of nail technicians to VOCs would be among the major factors probably thought of as being responsible for this kind of differences. But first, it has to be stated, that based on the maximum combined exposure to the mixture of VOCs observed in our study, which was below 20 % and 25 % of TWA-OEL during the “hot” and “cold” season, respectively, we do not have any reasonable evidence to conclude that this occupational group is overexposed to VOCs, at least as long as the current occupational standards are considered. This conclusion seems to hold true also when occupational exposures to individual VOCs investigated in our study are considered separately: since the airborne levels of all VOCs investigated in our study were all far below the respective TWA-OELs, no overexposure to any VOC investigated in this study can be assumed. Similar outcomes were reported previously by several other studies [17, 20, 36, 41–43].

Even though the occupational exposure to VOCs among nail technicians was found to be very low, we found it to be significantly increased during the “cold” season compared to the “hot” one: the differences were apparent in the case of the combined exposure to the mixture of VOCs (3.0-times higher during the winer; *p* < 0.0005), as well as in the case of the airborne levels of the three most abundant VOC found in workroom air samples - ethanol (4.4-times higher; *p* < 0.0001), 2-propanol (2.6-times higher; *p* < 0.01) and ethyl acetate (2.9-times higher; *p* < 0.05). A question arises as what can be the cause of such increased airborne levels of organic solvents in nail salons during the “cold” season. Available studies indicate that in occupied houses, the ventilation behavior varies throughout the year and that it may significantly influence the rate of air exchange in rooms [44]. Moreover, studies by Goldin et al. [42] and Reolofs et al. [20] provide evidence of increased CO_2_ content in some 75 % of nail salons indicating that the vast majority of nail salons do not meet the minimum ventilation requirements. Even though no records of window opening or any ventilation measurements in nail salons were performed in this study, based on this data, one can now speculate that the pattern of seasonal variation of the levels of biomarkers of oxidative stress found in nail technicians enrolled in our study may be attributable to their increased occupational exposure to VOCs due to limited workroom ventilation during the “cold” season and that this effect might have outmatched the effects supposedly imposed by increased temperature, humidity and sunlight during the “hot” season, observed among control subjects. This hypothesis, however, seems not to be supported by the outcomes of the correlation analysis performed in this study, as this analysis provided very limited evidence corroborating the possible correlation between the airborne levels of VOCs and the levels of biomarkers of oxidative stress among nail technicians. The airborne ethanol and ethyl acetate, the two of three most abundant VOCs, were the only exception, as they were found to be positively correlated with the activity of GPx1 and blood plasma levels of Cp, respectively. Nevertheless, these correlations, despite being statistically significant (*p* < 0.05), were relatively weak, explaining only some 7 % and 5 % of the seasonal variability of the respective biomarker in nail technicians. This may indicate, that the background underlying the seasonal changes observed in nail technicians might be more complex than just simple associations with airborne levels of VOCs during work-time and that possible differences in some other factors (including lifestyle-related factors, such as moderate physical activity, diet, coffee intake, alcohol consumption, etc.) not involved in this study may have contributed to observed outcomes. It might also be of importance, that airborne ethanol in nail salons was found to correlate significantly with the DNA strand breakage (r_SP_ = 0.287; R^2^ = 0.082; *p* < 0.001) and oxidatively generated DNA damage (r_SP_ = −0.259; R^2^ = 0.067; *p* < 0.05) in nail technicians ([13] and *unpublished data*), pointing to some plausible role of this organic solvent in predicting the level of investigated biomarkers among nail technicians. Although this compound has long been considered a potent inductor of reactive oxygen species both in vitro and in vivo [45], yet this aspect definitely requires more research.

Unfortunately, the number of studies investigating the effect of prolonged low-level occupational and/or the environmental exposure to VOCs on levels of biomarkers of oxidative stress is scarce so far, and they provide inconclusive outcomes. In studies involving occupationally exposed bus drivers, Rossner et al. failed to identify airborne VOCs as an independent factor predicting the levels of biomarkers of oxidative stress [39, 40]. On the other hand, low-level exposure to airborne toluene was found to be the only independent factor responsible for increased levels of biomarkers of oxidative stress and DNA damage among painters exposed to various xenobiotics in paints [9, 10]. In general population, the low-level environmental exposure to airborne VOCs was found to correlate significantly with biomarkers of oxidative stress (including DNA damage), although this correlation was limited to older subjects only [46]. It is therefore obvious, that this issue is not sufficiently understood and that further research is needed.

When interpreting our outcomes, one has to keep in mind, that only static and short-term breathing-zone area measurements were performed in order to assess the occupational exposure of nail technician, which might have led to slight underestimation of the real exposure. In addition to this, the measured exposures presented in our study need to be considered as approximations of the workday exposure to VOCs in nail salons, as they were averaged over 2 h on average only, not over a period of 6 h, which is required in Poland in order to make the reliable comparison with respective TWA-OELs. It also needs to be emphasized, that the “hot” and “cold” season groups investigated in our study consisted of unpaired subjects, what may have introduced some additional between-group variation. Although this variation was dealt with by using statistical methods suited for analysis of unpaired data (instead of methods developed for testing the significance of differences in paired data), this aspect still has to be kept in mind. Last but not least, no sunlight radiation, ambient temperature and humidity monitoring during the “hot” and “cold” season was conducted within the framework of our study, due to which we were unable to precisely infer how the occupational exposure to VOCs interfered with the effect of temperature, humidity and sunlight radiation and/or their variations between the two seasons under discussion.

## Conclusion

To sum up, in this study we provide evidence, that nail technicians present significantly different pattern of seasonal variation in selected biomarkers of oxidative stress compared to control subjects. Although such differences might intuitively be attributed to occupational exposure of nail technicians to VOCs which we found to be higher during the “cold” season compared to the “hot” one, our study did not provide convincing evidence in favor of such hypothesis and thus further studies must be conducted in order to elucidate possible factors underlying such differences between control subjects and nail technicians observed in our study.

## Abbreviations

% DNA, relative amount of DNA in the comet tail; ACGIH, American Conference of Governmental Industrial Hygienists; ALSs, alkaline-labile sites; Cp, ceruloplasmin; FPGs, formamidopyrimidine glycosylase-sensitive sites; GPx, glutathione peroxidase; IARC, International Agency for Research on Cancer; IQR, Interquartile Range; MANCOVA, multivariate analysis of covariance; PY, pack-years; RBCs, red blood cells; SD, standard deviation; SOD1, zinc-copper superoxide dismutase; SSBs, single strand DNA breaks; TBARS, thiobarbituric acid-reactive species; TWA-OEL, time-weighted average occupational exposure limit; VOCs, volatile organic substances

## Additional file

Additional file 1: Dataset. (XLSX 57 kb)
